
JOURNAL INFORMATION
==============================
NLM Title Abbreviation: Tex Med J (Austin)
NLM Journal ID: 21130590R

Texas Medical Journal

ISSN: 0892-8495
Publisher: Biomedical Journal Digitization Project

ARTICLE INFORMATION
==============================
PMCID: PMC9615810
Article version: 1
Subjects: Books and Magazines

International Clinics

A Quarterly of Clinical Lectures and Especially Prepared Articles on Medicine, Neurology, Surgery, Therapeutics, Obstetrics, Pediatrics, Pathology, Dermatology, Diseases of the Eye, Ear, Nose and Throat, and Other Topics of Interest to Students and Practitioners

Print publication date: 1902 Apr
Volume: 17
Issue: 10
First page: 391
Last page: 391
License: This work is free of known copyright restrictions. For more information, please see PMC Back Issue Digitization.
License URL: https://creativecommons.org/publicdomain/mark/1.0/

==============================

Phillips' Emulsion 50% best NORWAY COD LIVER OIL minutely sub-divided, WITH WHEAT PHOSPHATES (Phillips')

Pancreatized, Palatable, Permanent, Miscible in Water, Milk, Wine, etc.

Phillips' Milk of Magnesia $Mg H_2 O_2$ (FLUID.) "THE PERFECT ANTACID."

for correcting Hyperacid conditions—local or systemic.
Vehicle for Salicylates, Iodides, Balsams, etc.

Phillips' Phospho-Muriate of Quinine, COMP.

TONIC AND RECONSTRUCTIVE.

WHEAT PHOSPHATES, WITH MURIATE OF QUININE AND STRYCHNINE.

PHILLIPS' WHEAT PHOSPHATES (ACID).
PHILLIPS' SYRUP OF WHEAT PHOSPHATES.
PHILLIPS' DIGESTIBLE COCOA.

THE CHAS. H. PHILLIPS CHEMICAL CO., NEW YORK

International Clinics.—A quarterly of clinical lectures and especially prepared articles on medicine, neurology, surgery, therapeutics, obstetrics, pediatrics, pathology, dermatology, diseases of the eye, ear, nose and throat, and other topics of interest to students and practitioners. By leading men of the medical profession throughout the world. Edited by Henry W. Cottell, M. D., A. M., Philadelphia, with the colaboration of John B. Murphy, M. D., of Chicago; A. D. Blockader, M. D., of Montreal; H. C. Wood, M. D., of Philadelphia; T. M. Rotch, M. D., of Boston; E. Landolt, M. D., of Paris; Thos. G. Morton, M. D., of Philadelphia; C. H. Reed, M. D., of Philadelphia; J. W. Ballantyne, M. D., of Enburgh, and John Harold, of London. With regular correspondents in Montreal, London, Paris, Leipsig and Vienna. Vol. IV; eleventh series, 1902. Philadelphia: J. B. Lippincott Company. 1902. Price, $2.00 per volume.

These volumes are invaluable to the general profession. This is one of the most interesting volumes that has been issued. It contains one hundred and seven illustrations, eighty-four special formulas and a table on infant feeding from one week to three years. There are thirty-six authors who contributed to this volume. This is only one of the four volumes the subscriber gets during the year.

T. J. B.
